# The Role of HDL and HDL Mimetic Peptides as Potential Therapeutics for Alzheimer’s Disease

**DOI:** 10.3390/biom10091276

**Published:** 2020-09-04

**Authors:** Dustin Chernick, Rui Zhong, Ling Li

**Affiliations:** 1 Graduate Program in Pharmacology, University of Minnesota, Minneapolis, MN 55446, USA; dustin.chernick@gmail.com; 2 Department of Experimental and Clinical Pharmacology, University of Minnesota, Minneapolis, MN 55446, USA; zhong355@umn.edu

**Keywords:** high-density lipoproteins, apolipoproteins, mimetic peptides, Alzheimer’s disease

## Abstract

The role of high-density lipoproteins (HDL) in the cardiovascular system has been extensively studied and the cardioprotective effects of HDL are well established. As HDL particles are formed both in the systemic circulation and in the central nervous system, the role of HDL and its associated apolipoproteins in the brain has attracted much research interest in recent years. Alzheimer’s disease (AD) is the most prevalent neurodegenerative disorder and the leading cause of dementia worldwide, for which there currently exists no approved disease modifying treatment. Multiple lines of evidence, including a number of large-scale human clinical studies, have shown a robust connection between HDL levels and AD. Low levels of HDL are associated with increased risk and severity of AD, whereas high levels of HDL are correlated with superior cognitive function. Although the mechanisms underlying the protective effects of HDL in the brain are not fully understood, many of the functions of HDL, including reverse lipid/cholesterol transport, anti-inflammation/immune modulation, anti-oxidation, microvessel endothelial protection, and proteopathy modification, are thought to be critical for its beneficial effects. This review describes the current evidence for the role of HDL in AD and the potential of using small peptides mimicking HDL or its associated apolipoproteins (HDL-mimetic peptides) as therapeutics to treat AD.

## 1. Introduction

Alzheimer’s disease (AD) is a progressive, age-related neurodegenerative disorder. AD is characterized by the presence in the brain of amyloid-β (Aβ) deposited in senile plaques and cerebral vessels, neurofibrillary tangles, and eventually the loss of neurons [1]. AD is the most common cause of dementia in the elderly, and affects over 5 million people in the United States and around 50 million people worldwide [2,3]. Alarmingly, the number of people with AD is predicted to triple by the year 2050, as our population continues to age. While aging is the primary contributor to the development of AD, inheritance of the apolipoprotein (APO) E4 gene was identified as the strongest genetic risk factor for sporadic, late onset AD [4,5,6], which has been confirmed by meta-analyses of many studies [7,8,9]. As a major apolipoprotein involved in maintaining lipid/cholesterol homeostasis and cardiovascular health, APOE has ignited research into the connections between cardiovascular disease and AD, and raised the possibility that therapeutic strategies developed for preventing atherosclerosis may be repurposed to mitigate AD.

With no drugs currently proven to stop, or even slow, the progression of AD, this disease represents one of the largest unmet medical needs today. Of the five drugs approved to treat AD, four share the same mechanism of action (acetylcholinesterase inhibition), and all provide only modest symptomatic benefits with uncertain long-term efficacy and safety [10,11]. The drug most recently approved by the US Food and Drug Administration (FDA) for the treatment of AD was in 2004. Although almost all clinical trials for AD have failed since then, increased government and societal support for basic research in AD has led to significant advances in our understanding of molecular pathways related to AD risk and progression, and a slew of novel agents in different stages of clinical development targeting pathways including Aβ, tau, and inflammation [12].

This review focuses on the role of high-density lipoproteins (HDL), commonly referred to as the “good cholesterol”, which has a wide range of well-known cardioprotective and anti-inflammatory functions [13], and the therapeutic potential of HDL-based strategies. We highlight the evidence supporting a critical role of vascular health in the prevention and treatment of AD, and propose that small peptides designed to mimic the beneficial properties of HDL (HDL-mimetic peptides) are an attractive therapeutic avenue in AD, and an important area for future research and development efforts.

## 2. Overview of HDL Structure and Function in the Systemic Circulation and in the Central Nervous System (CNS)

Lipoprotein is a molecular complex of proteins and lipids. An inner hydrophobic core and an outer hydrophilic shell constitute the general spherical structure of mature lipoprotein particles. Among all lipoproteins, HDL has the smallest diameter (7.2–13 nm) and largest density (1.063–1.21 g/mL) [14]. HDL contains a plethora of lipid and protein components and thus exhibits multiple functions. The lipid composition of HDL is predominantly populated by phospholipids (PL), sphingomyelins (SM), and neutral lipids including cholesteryl esters (CE), unesterified free cholesterol (FC), and triglycerides (TG). The hydrophobic core is composed primarily of TG and CE, whereas PL and FC make up the hydrophilic shell. The outer shell is also constituted by apolipoproteins (APOs) including APOA-I, APOE, APOD, and APOJ (also known as clusterin). Multiple other proteins such as enzymes, lipid transferring proteins, and proteinase inhibitors are also present in HDL [14]. These lipids and proteins mediate HDL interactions with a variety of other molecules including enzymes, transporters, and receptors through a dynamic process.

The protein components, especially APOs, largely determine the key functions of HDL. Among them, APOA-I is the principal protein component that explains the structural and functional aspects of HDL in the systemic circulation [15]. The 243-residue polypeptide in human is mainly expressed in liver and intestine [16]. In plasma, more than 90% of APOA-I is in the form of lipoprotein whereas lipid-poor/free APOA-I only accounts for around 5% which, however, is highly metabolically reactive in accepting cell cholesterol and PL and ready to form lipoproteins [17]. The residues 44–243 of APOA-I is the lipid-associating domain featuring amphipathic α-helical structural repeats [18]. APOA-I is the primary acceptor of cell cholesterol and phospholipids via the interaction with the plasma membrane mediated by the ATP-binding cassette transporter ABCA1 [15]. These functional amphipathic α-helices have also been found in other APOs and similar sequential binding process has been reported [19,20]. For example, APOE also has a major lipid-binding domain with a series of α-helices close to the C-terminus in addition to its LDL receptor-binding region near the N-terminus. Human APOE has 299 amino acids, and is synthesized predominately by the liver (>75%) and the brain [21]. Unlike APOA-I, which is produced primarily in the periphery and only limitedly reaches the CNS by crossing the blood–brain barrier (BBB) or the blood–cerebrospinal fluid barrier (BCSFB) at choroid plexus [22,23,24], APOE is the main APO in the brain. Notably, APOE in the periphery does not cross the BBB and thus, APOE in the brain is exclusively synthesized locally, predominantly by astrocytes, and forms HDL-like particles [25]. APOE exists in three isoforms in human: APOE2, APOE3, and APOE4 with the APOE3 being the most prevalent. Amino acid sequencing uncovered that the three isoforms differ by one amino acid at either position 112 or 158 [26,27]. Genome-wide association studies (GWAS) have identified the APOE-ε4 allele, which encodes the APOE4 isoform (Arg112 and Arg158), as the strongest genetic risk factor in AD [4,5,6]. The connection between APOE and AD will be discussed later in greater detail. Another major HDL-associated APO is APOJ, which is also known as clusterin (CLU) in the CNS. Encoded by the single-copy CLU gene located on chromosome 8 [28], APOJ is a 427-amino-acid polypeptide primarily expressed in the liver, brain, ovary, and testes with two subunits (α- and β-CLU) and repeats of α-helices [29]. APOA-I, APOE, and APOJ, along with other molecules, regulate the complex structural and functional aspects of HDL.

The physiological association between plasma HDL and cardiovascular disease has been well described previously [13]. The underlying cause of cardiovascular disease is atherosclerosis, which is driven by the retention of APOB-associated lipoproteins (majorly LDL) along the vessel wall, leading to endothelial dysfunction and subsequent infiltration of more APOB-containing lipoproteins. This initiating event further sparks a series of sequential inflammatory responses including macrophage recruitment, oxidative stress, and foam cell formation. Importantly, while high levels of LDL are atherogenic, HDL can remove excess cholesterol to prevent plaque formation by reverse cholesterol transport (RCT), in which HDL collects cholesterol from cells via ABC transporters such as ABCA1, ABCG1 and ABCG4, and transports cholesterol from peripheral tissues back to the liver for biliary excretion [30]. HDL also mitigates atherosclerosis via various other pathways including anti-oxidative, anti-inflammatory, pro-endothelial and vasodilatory, antithrombotic, and immune-modulatory mechanisms [13,31].

**Figure 1:**
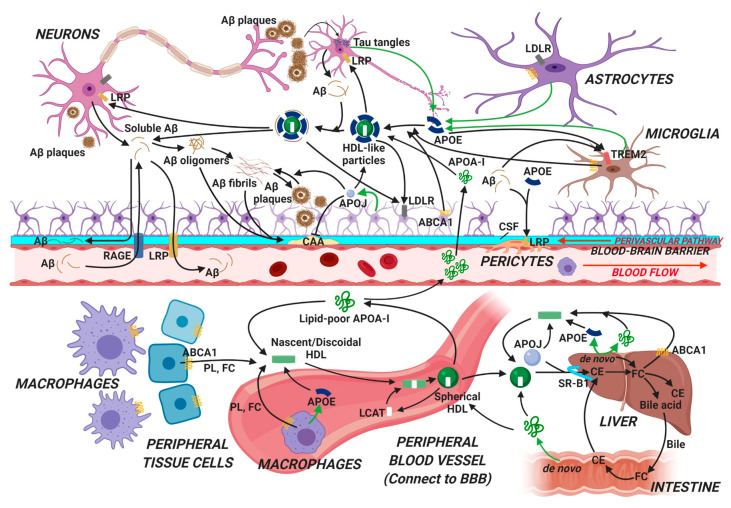
Schematic summary of apolipoproteins/high-density lipoproteins (APOs/HDL) metabolic pathways in the periphery and the brain pertinent to Alzheimer’s disease (AD). In the periphery, the major APO is APOA-I that is produced by the liver and the intestine, and subsequently binds to lipids to form lipoprotein particles including HDL along with other APOs. APOs/HDL interact with ABCA1 and remove excess cholesterol and phospholipids from tissues via reverse cholesterol transport, and bring them back to liver for biliary excretion. In the brain, the main APO is APOE that is produced primarily by astrocytes. A limited amount of APOA-I in the plasma can cross the blood–brain barrier (BBB) into the brain. APOs bind to lipids and form HDL-like particles, similar to HDL formation in the periphery. APOs/HDL interact with different cell types in the brain and regulate Aβ aggregation/clearance and tau tangle formation in AD (see the main text for details). HDL, high-density lipoprotein; PL: phospholipids; FC, free cholesterol; CE: cholesterol ester; CSF, cerebral spinal fluid; CAA, cerebral amyloid angiopathy; ABCA1: ATP-binding cassette transporter A1; LCAT, lecithin–cholesterol acyltransferase; SR-B1: scavenger receptor B1; LDLR, low-density lipoprotein receptor; LRP, low-density lipoprotein receptor-related protein; RAGE: receptor for advanced glycation endproducts; TREM2: triggering receptor expressed on myeloid cells 2. Figure created with BioRender.com.

In addition to cardiovascular disease, there is increasing interest in the connection between HDL and CNS diseases. Despite being separated into two distinct pools by the BBB and the BCSFB, HDL in the periphery and HDL-like particles in the CNS share similar roles in maintaining homeostasis of lipid metabolism and preserving vascular functions [25]. As the major APO in the brain, APOE plays a central role in HDL metabolism and function in the CNS under physiological and pathological conditions (Figure 1).

## 3. Role of HDL in AD

Mounting evidence points towards a pivotal role of HDL in AD [32,33]. The main pathological hallmarks of AD include the extracellular neuritic plaques formed by accumulation of Aβ, and neurofibrillary tangles (NFTs) constituted by hyperphosphorylated tau proteins in the brain [1]. The precise mechanisms by which HDL influences the development of AD are not completely understood; however, HDL-associated APOs that define HDL functions substantially modify the risk of AD. This section aims to provide an overview of studies in support of the important role of HDL and associated APOs in AD from multiple lines of epidemiological/clinical, genetic, and biochemical evidence.

### 3.1. Epidemiological and Clinical Evidence

Extensive epidemiological and clinical research has documented a protective role of HDL in reducing the risk of AD. For example, recently reported results from the Baltimore Longitudinal Study of Aging, which followed 889 participants for 20 years, found a positive association between HDL-C levels and brain volume, and that cognitively impaired participants at follow-up had lower baseline HDL-C levels [34]. The results are consistent with earlier reports that AD patients had lower plasma levels of HDL-C and APOA-I, when compared to healthy controls, and that both HDL-C and APOA-I levels inversely correlated with cognitive impairment [35,36]. Additionally, an inverse relationship between HDL levels and AD risk was observed in studies conducted across different study sites and ethnic groups as summarized in Table 1.

However, there have been several studies that reported findings contradictory to those described above. Some showed that high levels of HDL-C correlated with cognitive decline and more severe AD pathology [51,52]. This inconsistency may be explained in part by the different age groups of participants in these studies. Studies that observed a negative impact of HDL on AD were conducted amongst older participants, when substantial neuropathology and cognitive impairment may have already occurred. Furthermore, other metabolic disorders, such as diabetes the risk of which rises significantly with age, may confound assessments in older subjects [46,53]. Moreover, some studies indicated no association between HDL-C and cognitive function, including earlier studies [54,55] and some more recent studies [56,57]. It is worth noting that HDL may serve as a “double-edged sword”; under normal physiological conditions, HDL exerts anti-inflammatory effects; whereas under some pathological conditions, HDL may become dysfunctional and pro-inflammatory [58]. In addition, while the level of HDL-C provides an estimate on the quantity of HDL, it does not predict the quality of HDL. The function of HDL in the systemic circulation is mainly determined by the level and state of APOA-I [32]. This may explain some of the discrepancies in clinical studies when only levels of HDL-C were used to determine the association of HDL with disease status.

### 3.2. Genetic Evidence

Alzheimer’s disease is highly heritable (estimated at ~80%) [59] and is genetically dichotomous, featuring both a familial early-onset and a sporadic late-onset form, with the latter accounting for over 95% of all AD cases. The earliest genetic evidence supporting the connection between HDL and AD comes from the discovery of the APOE-ε4 allele as a major risk factor for AD, associated with both familial and sporadic AD in a dose-dependent manner [4,5,6,60]. Two copies of the *ε4* allele (homozygous) increase sporadic AD risk by 15-fold, whereas a single allele (heterozygous) contributes to a 3-fold increase in risk [61]. The allele frequencies of ε2, ε3, and ε4, are 7%, 78%, and 15%, respectively, in most Caucasian populations [62], showing that APOE3 is the most common isoform in humans and APOE2 is the least common. However, in patients with AD, the frequency of the APOE-ε4 allele is markedly increased to 40–50% [60]. Opposite to APOE-ε4, APOE-ε2 confers a reduced risk of AD [4,5,6], although APOE-ε2 is associated with more severe CAA and hemorrhages [63,64]. Among all three APOE isoforms, APOE2 and APOE3 are preferentially associated with HDL while APOE4 with LDL and VLDL, lipoproteins that are strongly associated with cardiovascular diseases and AD [65,66]. This provides a possible reason why APOE-ε4 links to high AD risk from a cholesterol removal standpoint. Notably, the pleiotropic APOE is such a strong risk factor that its association with AD and HDL individually has achieved genome-wide significance in the single-trait GWAS [67,68].

In addition to the three common alleles of APOE, rare mutations in APOE also influence the risk of AD. For instance, the APOE4 Pittsburgh mutation (p. L28P) was reported to be associated with a further increase in risk for late onset AD [69], whereas the APOE3 variant p.V236E was found to be associated with a markedly reduced risk of AD [70]. Recently, a case report showed that a rare APOE3 variant, APOE-Christchurch (p. R136S), in homozygosity, was associated with a dramatic delay in the development of AD in an individual carrying the familial PSEN1 mutation [71]. Intriguingly, this individual has a massive buildup of amyloid plaques in the brain but with a surprisingly low level of tau and neurodegeneration. The authors suggested that one possible explanation is that the Christchurch mutation prompts a weak affinity of APOE to heparan sulfate proteoglycans (HSPG), a glycoprotein that plays a crucial role in the spread and uptake of toxic forms of tau [72,73]. Although the exact mechanisms underlying such marked resilience to AD remain evasive, this report is expected to harbinger future research on the role of APOE/HDL in the pathogenesis of AD and its therapeutic implications.

Other HDL-related genes that have been recognized as risk factors for AD are also gaining increasing attention. Meta-analyses of GWAS data have identified more than 20 loci that contribute to the risk of sporadic AD [7,8,9]. Importantly, the lipid metabolism pathway has been consistently implicated in the development of AD. In addition to *APOE*, *CLU,* and *ABCA7*, well-known genes that are intimately involved in lipid/cholesterol metabolism and AD pathogenesis, many other genes involved in protein-lipid complex assembly, reverse cholesterol transport, and plasma lipoprotein/HDL particle assembly pathways have been shown to influence the risk of AD [7,8,9]. Notably, the HDL-related genes *APOM*, *APOA5*, and *ABCA1* were found to drive the association of the lipid metabolism pathway with AD risk. Further, an HDL-C and TG-associated gene, *WWOX,* which is highly expressed in astrocytes and neurons, was identified as a new risk locus for late-onset AD, likely through its interaction with tau. Taken together, these findings further reinforce the connections between HDL-related pathways and AD.

### 3.3. Biochemical Evidence in Preclinical Models

There has been a tremendous increase in preclinical and biochemical evidence for the role of HDL in AD in recent years. With both in vitro and in vivo models, experimental investigations have demonstrated that HDL/APOs modulate different aspects of AD pathologies.

#### 3.3.1. Amyloid Pathology

The amyloid pathology of AD is characterized by the deposition of Aβ in the brain parenchyma forming senile plaques (amyloid plaques), and in the cerebral vasculature leading to cerebral amyloid angiopathy (CAA). Aβ is generated through the amyloidogenic pathway in which the transmembrane amyloid precursor protein (APP) is sequentially cleaved by β-secretase (also known as β-APP cleavage enzyme 1, BACE1) and γ-secretase [74]. The protective non-amyloidogenic pathway, on the other hand, features initial cleavage by α-secretase within Aβ sequence, preventing the formation of intact Aβ. Aβ40 and Aβ42 are the two main products from the amyloidogenic pathway, with Aβ40 in greater abundance and Aβ42 more prone to aggregation. Whereas Aβ42 is the predominant species in parenchymal amyloid plaques, Aβ40 is the main constituent of CAA, although Aβ42 is essential for initiating both parenchymal and vascular amyloid deposition [75].

HDL/APOs influence amyloid pathology through a variety of mechanisms, most notably APP trafficking/processing and Aβ production/clearance pathways. APOE is the most extensively investigated HDL-associated APO in modulating amyloid pathology, which has been thoroughly reviewed recently [25,76,77,78]. In brief, APOE has been reported to participate in all aspects of APP/Aβ metabolism in the brain, including APP processing and Aβ generation, Aβ aggregation, intracellular and extracellular Aβ degradation, and Aβ clearance through the BBB and lymphatic pathways. Studies have shown that the effects of APOE on Aβ pathology are mediated through direct or indirect interactions and are dependent on the isoform, the level, and the lipidation status of APOE. Compared with APOE2 and APOE3, APOE4 exists in lower levels, is less lipidated, and is associated with increased Aβ production/aggregation and decreased clearance. The majority of these findings have been obtained by studying human *APOE* isoform-specific targeted replacement (TR) mice crossed with transgenic AD mouse models. Recently, to address the role of peripheral and central APOE, respectively, in AD, novel lines of human APOE-knock in (KI) mice have been generated [79]. It was shown that deletion of hepatic *APOE*, which led to a marked decrease in peripheral APOE levels, did not affect cerebral Aβ deposition in the APP/PS1 transgenic mouse model of AD. Interestingly, earlier studies had reported that expression of APOE only in the periphery exerted beneficial synaptic/cognitive effects in APOE-deficient mice [80], and that such benefits were observed only when APOE3, but not APOE4, was expressed in the liver [81]. A recent study in a bioengineered human cerebral vessel model also showed that luminal APOE-enriched HDL particles reduced CAA, indicating protective effects of peripheral APOE against vascular Aβ deposition [82]. Thus, the overall impact of peripheral and central APOE in AD remains to be defined.

A growing body of evidence suggests that APOA-I also plays an important role in protecting against the development of amyloid pathology. Animal studies have illustrated that a lack of APOA-I exacerbates—while overexpression of human APOA-I alleviates—CAA in the APP/PS1 mouse model of AD, without affecting parenchymal Aβ deposition [83,84], consistent with a prior study in a different line of APOA-I-deficient AD mice (PD-APP) [85]. On the contrary, a recent study using another transgenic mouse model of AD (Tg2576) showed that lack of APOA-I decreased both parenchymal and vascular Aβ pathology [86]. However, another recent study with APP/PS1 mice extended prior findings, in which APOA-I deficiency increased CAA and neuroinflammation as well as cortical Aβ deposition [87]. The differences between the AD animal models used and the ages of the mice studied may explain these discrepancies. In vitro studies have demonstrated that APOA-I binds to Aβ, inhibits Aβ aggregation, and alleviates cytotoxicity [88,89], supporting a protective role of APOA-I in β-amyloidosis. Moreover, a single intravenous injection of reconstituted HDL decreased both soluble Aβ40 and Aβ42 levels in the brain after 24 h in APP/PS1 mice [90]. Recent in vitro studies reinforce the role of APOA-I in facilitating Aβ clearance. In a cellular model of the BBB, luminal (blood) APOA-I, as well as APOJ, mobilized the abluminal (brain) efflux of Aβ across cerebrovascular endothelial cell monolayers [91]. In addition, as with APOE, the lipidation state of APOA-I was found to robustly influence Aβ efflux across the BBB model [92]. The ability of APOA-I to promote Aβ efflux increased alongside its lipidation status and reached maximum Aβ efflux when APOA-I was present in discoidal HDL particles. In a bioengineered human cerebral vessel model, purified, APOA-I-enriched plasma HDL suppressed Aβ-induced peripheral blood mononuclear cell (PBMC) adhesion to human endothelial cells through SR-BI [93]. The same group also found that APOA-I-enriched circulating HDL and APOE (in an isoform-dependent manner) synergized to boost Aβ efflux across the bioengineered human cerebral vessels. This ability of APOA-I to improve clearance of Aβ out of the brain and into the blood, where it is readily degraded, may represent a key mechanism by which it enacts its protective effects in AD.

The implication of APOJ in Aβ fibrillization and clearance in the context of AD has also been investigated. Early studies in vitro and in vivo demonstrated that APOJ inhibits Aβ aggregation [94,95], enhances phagocytosis of Aβ aggregates [96], and facilitates the clearance of Aβ across the BBB [97]. Using various in vitro BBB models, recent studies have confirmed the role of APOJ in modulating Aβ aggregation and clearance [91,98,99]. In addition, lack of APOJ was found to shift Aβ deposition to the cerebrovasculature, disrupt perivascular drainage pathways, and lead to severe CAA in APP/PS1 mice [100], providing in vivo evidence for the role of APOJ in the brain vascular clearance of Aβ [101]. Consistently, a recent study showed that intravenous administration of human recombinant APOJ reduced insoluble Aβ levels and CAA in the brain of APP23 mice [102], raising the possibility of elevating plasma APOJ levels to mitigate Aβ pathology in AD.

#### 3.3.2. Tau Pathology

Tau is a microtubule-associated protein that becomes aberrantly hyperphosphorylated, dissociates from microtubules and then aggregates during AD, forming intracellular neurofibrillary tangles, which ultimately leads to neurodegeneration [103]. Recent studies have shown that HDL/APOs modulate tau pathology, in addition to Aβ pathology, synergistically or independently. APOE and its isoforms remain the most heavily explored HDL components in tauopathy in the context of AD, although both CSF APOA-I and APOJ levels have been associated with CSF tau or p-tau levels [104,105]. An early study demonstrated that the accumulation of toxic APOE4 fragments thorough neuron-specific proteolysis increases tau phosphorylation in brains of transgenic mice [106]. Further investigations came from tau/APOE models that were generated by either crossing the tauopathy P301S mouse model with or AAV gene delivery of P301L tau into human APOE-TR models that are isoform specific [107,108]. The former study revealed that APOE-KO was more neuroprotective against tau-mediated neurodegeneration than APOE3 or APOE2, while *APOE4-TR* led to marked exacerbation of tau pathology. Interestingly, contradictory results were seen in the latter study, in which tau pathology and behavioral deficits were more severe in APOE2-TR mice than APOE3-TR and APOE4-TR. The discrepancies in results are possibly due to the different experimental model systems utilized in the two studies, which can result in disparate features including total and p-tau expression levels, toxicity of the two tau species, and age-related tau aggregation [108]. Of note, the effects of APOE alleles on tauopathies can be modified by the presence of amyloid pathology [108,109], indicating an intricate relationship between the two pathological hallmarks of AD. Intriguingly, several lines of evidence have also suggested that tau and Aβ pathologies are driven through independent yet common upstream pathways involving cholesterol metabolism and APOE [110,111,112,113].

Advances in induced pluripotent stem cell (iPSC) technologies have allowed for the study of endogenous tau regulation in functional human neurons from both AD patients and healthy controls. In human iPSC-derived APOE4-expressing neurons, tau phosphorylation as well as Aβ production was elevated when compared to those in isogenic neurons expressing APOE3 [113]. The detrimental effects of APOE4 were also shown in human iPSC-derived brain organoids as well as individual cell types [114]. Mechanistically, APOE4 has been reported to accelerate the spreading of tau pathology and neuron death in part by neuron-specific, glia-independent pathways [115]. Intriguingly, CE, the storage form of excess cholesterol, was found to increase p-tau accumulation in iPSC-derived AD neurons independent of its effects on Aβ [110], suggesting that enhancing HDL/APO-mediated cholesterol efflux to reduce cellular CE content could potentially mitigate p-tau accumulation in the brain.

#### 3.3.3. Neuroinflammation

The innate immune response in the brain, primarily mediated by microglia and astrocytes, plays an essential role in AD pathogenesis. Cerebral amyloid pathology, tau pathology, or both are often associated with concomitant neuroinflammation, in which APOE is a crucial player. Recent studies show that APOE exerts its neuroimmune modulatory function partly through interactions with the triggering receptor expressed on myeloid cells 2 (TREM2), following the discovery that mutations in TREM2 were associated with increased risk of AD [116,117]. In the brain, TREM2 is exclusively expressed by microglia. The role of TREM2 in AD has been extensively reviewed [118,119,120,121], in particular the impact of APOE-TREM2 interactions on AD-related neuroinflammation thoroughly covered [25,122]. In brief, as a ligand for TREM2, APOE interacts with TREM2 and drives dysfunctional phenotypes of microglia in aging and in the pathological process of AD. TREM2-deficient/mutant microglia fail to surround and clear Aβ plaques and modulate tau pathology and associated neuroinflammation and neurotoxicity. More recently, it has been shown that microglia drive tau pathology and neurodegeneration depending on APOE [123]. It also appears that the inflammatory response in the brain is regulated specifically by microglia-derived APOE [79]. In addition, TREM2 has been shown to regulate cholesterol metabolism in microglia, particularly upon chronic phagocytic challenge, and TREM2-deficient microglia fail to upregulate lipid metabolism genes including APOE, leading to intracellular accumulation of cholesteryl esters, which is rescued by treatment with pharmacological agents to inhibit CE synthesis or increase expression of cholesterol efflux genes [124]. Notably, APOE deficiency also causes CE accumulation in the brain, including both microglia and astrocytes, suggesting that loss of function in TREM2 triggers cholesterol transport defects and sequelae in microglia.

Besides APOE, other APOs and lipoprotein particles along with negatively charged lipid molecules have also been shown to be putative ligands for TREM2 [121,125]. Interestingly, while APOE and APOJ bind to TREM2 only when lipidated, APOA-I binds to TREM2 in either lipidated or non-lipidated forms [125]. To what extent these interactions affect the pathogenic process of AD remains to be investigated.

In addition to APOs, other HDL-associated components contribute to the anti-inflammatory and anti-oxidative properties of HDL. In particular, paraoxonase 1 (PON1) is a major HDL-associated enzyme in the plasma, which plays a crucial role in protecting against the atherogenic effects of oxidative stress on the vasculature [126]. Interested readers are referred to the excellent review on the potential role of PON1 in AD [127]. Recent advances have also highlighted the contribution of oxidized cholesterol metabolites, referred to as oxysterols, to AD pathogenesis. Unlike cholesterol, oxysterols can cross the BBB from the brain to the circulation and vice versa, and the two main oxysterols are 24-hydroxycholesterol produced in the brain and 27-hydroxycholesterol primarily produced in the periphery [128,129]. Numerous studies have shown that oxysterols modulate the progression of AD through multiple pathways, including neuroinflammation, amyloid pathology, and neuronal death, which have been discussed in detail in comprehensive reviews [130,131,132,133].

#### 3.3.4. Cerebrovascular Function

The vascular resilience associated with HDL has been investigated extensively in the periphery. HDL is known to exert vasoprotective functions via multiple mechanisms, and such protective functions can be impaired with age [134,135]. High level of HDL/APOA-I in the periphery is capable of clearing arterial plaques formed, in a large portion, by fat, cholesterol, and collagen. HDL, or its main protein component APOE in the CNS, is expected to have similar effects on cerebral vascular deposition of proteins.

To date, substantial evidence suggests that HDL protects against AD by improving BBB function. Recent studies using three-dimensional BBB models and neurovascular unit models have shown that HDL enhances vascular function and facilitates Aβ clearance from the brain, supporting a role for HDL in cerebrovascular resilience [82,93]. It is well known that CAA disrupts cerebral vascular integrity and impairs its function. APOA-I drives the critical role of HDL in cerebrovascular endothelial damage repair and inhibition of endothelial apoptosis [136]. Overexpression of human APOA-I mitigates CAA whereas lack of APOA-I exacerbates CAA in AD mouse models [83,84,87]. Moreover, pericytes, a key component in the BBB and the neurovascular unit, have recently emerged as a crucial regulator of vessel morphology and function [137,138]. Recently, APOE4 in pericytes was shown to contribute to cerebrovascular dysfunction by impairing pericyte-mediated basement membrane formation using both APOE-TR mice and primary pericytes [139]. In line with this study, others have shown basement membrane reduction in vessels in APOE-TR mice [140,141]. A previous study, however, reported that APOE4 produced by astrocytes, instead of pericytes, induces the reduction of tight junction proteins [142]. Further, another study using an in vitro BBB model showed APOE involvement in regulating tight junctions in an isoform-dependent fashion [143]. More recently, studies with APOE4 carriers show that APOE4 leads to BBB dysfunction and cognitive decline independent of AD-related neuropathology [144]. Together, these results indicate that APOs/HDL influence BBB integrity through a number of distinct mechanisms, mediated by different cell types within the neurovascular unit.

Additionally, the importance of CNS lymphatic vessels, located within the dura mater of the meninges, in AD pathogenesis has begun to gain attention recently [145]. Dysfunction of the meningeal lymphatic vessels has been implicated in AD pathogenesis, influencing Aβ clearance [146]. A recent study extends this knowledge by uncovering that the meningeal lymphatic vessels at the skull base are responsible for draining CSF, and this function is impaired with aging [147]. The nature of the lymphatic system suggests that this CNS lymphatic pathway would work closely in tandem with the innate immune system to modulate the elimination of toxic Aβ and other aberrantly aggregated proteins in AD brain. Notably, the HDL receptor SR-BI regulates perivascular macrophages and CAA in transgenic AD mice [148]. The potential of HDL and HDL-mimetic peptides in modulating meningeal lymphatic function awaits future investigation.

## 4. Potential of HDL-Based Therapies for AD

### 4.1. APOA-I Infusion and Recombinant HDL

Encouraged by the relationship observed between APOA-I levels in the plasma and reduced cardiovascular disease risk, in particular the low risk associated with a naturally occurring APOA-I variant (Milano) [149], a number of clinical trials have evaluated the safety and efficacy of APOA-I infusion in humans. Several groups have demonstrated the cardioprotective and anti-atherosclerotic potential of wild-type or Milano APOA-I—incorporated along with lipids into recombinant HDL (rHDL)—infusion [150,151,152]. However, recent randomized trials testing the infusion of rHDL particles with either wild-type APOA-I or APOA-I Milano failed to promote additional regression of coronary atherosclerosis in patients with intensive statin treatment [153,154]. These negative results have been questioned as these studies did not demonstrate an increase in quantity or quality of APOA-I/HDL by the treatment and that the subjects in these studies had a low-risk lipid profile, which might have masked the results of the trials [155]. Notably, a Phase III clinical trial is underway using human plasma-derived APOA-I and a new rHDL formulation to treat subjects with acute coronary syndrome (NCT03473223). This trial will help to establish the efficacy and safety profile of APOA-I in a patient population at risk of AD.

As for AD, both overexpression and infusion of human APOA-I have been shown to reduce neuroinflammation, inhibit cerebral amyloid angiopathy (CAA), and improve cognitive performance in transgenic mouse models [84,156]. However, whether infusion of full-length human APOA-I has any effect on human cognition has not yet been investigated. In the APP/PS1 transgenic mouse model of AD, a single intravenous injection of reconstituted HDL containing APOA-I and soy phosphatidylcholine was associated with reduced amyloid levels after one day, however, following four consecutive once-weekly injections, the improvement was no longer apparent [90]. Intriguingly, in the APP23 transgenic mouse model of AD, an eight-week chronic intravenous administration of recombinant APOA-I Milano led to a significant reduction in cerebral amyloid load, accompanied by reduced expression of neuroinflammatory markers [156]. Further, four-week administration of rHDL particles containing lipidated APOE3 increased Aβ clearance and boosted cognitive performance in a mouse model of AD [157]. These findings suggest that HDL-modulating strategies could mitigate AD as well as cardiovascular disease.

### 4.2. APO/HDL-Mimetic Peptides as Potential Therapeutics for AD

While the therapeutic potential of recombinant HDL-like particles in human neurological disease is an intriguing possibility, the clinical potential of full-length apolipoproteins is hindered by high costs associated with the mass-production, storage, and route of administration for a large protein with complex tertiary structure and post-translational modifications. In addition, as discussed above, HDL-associated APOs, including APOA-I and APOE, either have very limited or no penetrance across the BBB [22,158]. Small, orally bioavailable, BBB-penetrant peptides offer many advantages over their parent proteins when considering drug candidates.

First pioneered in the 1980s, HDL-mimetic peptides have been an area of interest in the cardiovascular field for some time [159]. These orally bioavailable small peptides, which retain the atheroprotective and anti-inflammatory effects of their parent proteins, have been developed to mimic specific apolipoproteins and typically retain the consensus sequences and/or structural similarities of key motifs, such as a lipid- or receptor-binding region [160]. Mimetics can be synthesized from D-amino acids and thus have higher oral bioavailability [161], and methods to improve oral delivery of these mimetics have been described [162]. Furthermore, HDL mimetic peptides have been shown to enter the brain [163] and cross the BBB substantially more efficiently than full-length APOs (Swaminathan et al. 2020; personal communication, manuscript in press). Due to their relative physiological contributions to HDL function, and the degree to which they have been studied, the most robust efforts have been focused on the creation of mimetic peptides derived from APOA-I, APOE, and APOJ. Other APOs have been used as a model for mimetics [164,165,166], but they have not been well studied.

#### 4.2.1. APOA-I Mimetic Peptides

Owing to the critical role of APOA-I in cardiovascular health, early efforts focused on developing mimetics of APOA-I. APOA-I mimetics are typically amphipathic by design, and adopt the alpha helical secondary structure of full-length APOA-I [16,159,160,167]. These peptides have been shown to bind lipids [168,169], and exert anti-atherosclerotic and anti-inflammatory properties [160,167]. Among the many variants created and tested, the most notable one is the peptide termed 4F, which is an 18 amino acid peptide containing four phenylalanine (F) residues [169,170]. 4F has been studied extensively in animal models and early human clinical trials for cardiovascular disease, using the peptide synthesized from L-amino acids (L-4F) or D-amino acids (D-4F). While preclinical studies consistently showed anti-atherosclerotic effects of 4F in multiple animal models [167,171], early human clinical trials have produced mixed results [172,173]. Recently, a human clinical trial showed that multiple oral doses of D-4F were well tolerated, which improved the HDL anti-inflammatory profile of human patients with cardiovascular disease [174]. Intriguingly, it was found that the oral dosage of 4F correlated more strongly with the extent of HDL anti-inflammatory capacity than did the plasma concentration achieved. Therefore, the authors argued that enteric exposure could be a critical factor in driving 4F-mediated beneficial effects. Supporting this idea, 4F was shown to promote RCT via trans-intestinal cholesterol efflux and mitigate intestinal inflammation [175,176].

Due to the well-established correlation between vascular risk factors and cognitive decline, APOA-I mimetic peptides have been tested for efficacy in improving cognitive performance. Oral D-4F combined with pravastatin inhibited Aβ plaque formation and improved cognitive function to a greater extent than pravastatin alone, in a mouse model of AD, by inducing an anti-inflammatory effect in the brain [177]. D-4F was not associated with changes in plasma HDL-C levels in this study, suggesting that the peptide improves HDL quality, not quantity, and/or directly modulates AD-related processes in the brain. In LDL receptor-null mice, oral D-4F reduced cerebrovascular inflammation and was associated with improved cognitive performance, without altering lipid levels in the plasma [178]. 4F was also found to protect against neuronal damage in a model of brain injury [179]. These studies indicate that the anti-inflammatory effects of 4F extend beyond the periphery into the brain, and suggest the potential for benefit in AD. Whether enteric exposure to 4F is important in mediating the AD-related effects of 4F has not been established.

In vitro studies of the 4F peptide in mouse and human primary glial cells demonstrated that 4F increases the lipidation of APOE, the primary apolipoprotein associated with HDL-like particles in the brain, and the greatest known genetic risk factor for AD. In this study, 4F was shown to counteract the inhibitory effects of aggregated Aβ, which reduces the secretion and lipidation of APOE [180]. This effect was shown to require ABCA1. Importantly, lipidation states of APOE regulate Aβ aggregation and degradation [181,182]. Compared with APOE2 and APOE3, APOE4 exhibits deficiency in lipidation, which is thought to drive the pathogenic effects of APOE on AD [181,182,183,184]. Thus, treatment with 4F may restore lipidation states of APOE4 and block a critical feed-forward cycle that leads to AD dementia. Our pilot studies support this notion [185,186].

#### 4.2.2. APOE Mimetic Peptides

As the most important and well-studied APO in the brain, APOE is an enticing target and the development of peptides to mimic its key functions has been undertaken by several groups. Mimetic peptides derived from the receptor-binding or lipid-binding regions of APOE have been developed and tested for neurological diseases including AD.

A novel APOE mimetic, COG1410 (derived from amino acids 138-149 of the APOE receptor-binding region), has been shown to improve cognitive function while reducing amyloid immunoreactivity and microglial activation after subarachnoid hemorrhage and traumatic brain injury (TBI) in mice [187,188,189]. This peptide crosses the BBB, and has been shown to exert its neuroprotective effects by reducing apoptosis and neuroinflammation [190], and by mitigating disruption of the BBB following experimentally-induced TBI [191]. COG1410 has also been shown to improve glucose uptake, reduce cerebral edema, and protect against neuronal atrophy in a model of TBI [192]. Vitek et al. examined the effects of this APOE mimetic in a mouse model of AD, and found that the peptide ameliorates behavioral deficits and reduces plaques and tangles [193]. COG1410 was recently shown to upregulate autophagy via phosphorylation of GSK3β [194]. Given the fact that GSK3β regulates tau phosphorylation, this finding may present one of the mechanisms underlying the effects of COG1410 on the tangle pathology.

Similar to COG1410, the peptide COG112 was derived from amino acids 133-149 of the receptor-binding region of APOE, which has been shown to be anti-inflammatory in mouse models of neurological disease [195,196,197,198]. Wang et al. described the ability of this peptide to mitigate elevation in brain amyloid levels following TBI [199]. Recently, COG112 was shown to rescue BBB function following traumatic spinal cord injury in APOE-knockout mice [200]. The potential of COG112 in AD was tested by Ghosal and colleagues, who found that the peptide reduced neuro-inflammation and protected against impairment of neurogenesis and tau pathology in an AD mouse model [201].

Another peptide containing this arginine-rich acetylcholine-inhibiting region of APOE is the dual peptide Ac-hE-18A-NH2, which contains residues 141–150 of APOE’s receptor-binding region covalently linked to the APOA-I mimetic 18A [202]. This peptide has been shown to be anti-oxidative, and vasculoprotective in vitro and in vivo [160]. The potential of this APOE/APOA-I mimetic in AD was tested in a mouse model of AD, and was demonstrated to improve cognition, decrease amyloid plaque deposition, and reduce glial activation [203]. While that study observed enhancement of monocytic Aβ uptake by Ac-hE-18A-NH2, this peptide was recently shown to inhibit astrocyte-mediated uptake of Aβ, and to reduce Aβ-induced pro-inflammatory cytokine release [204]. Importantly, this dual APOE/APOA-I peptide employs 18A as the APOA-I component. Given that 4F has superior lipid-binding affinity compared to 18A [169], it would be interesting to determine whether combining 4F with the receptor-binding region of APOE, to create Ac-hE-4F-NH2, produces more robust protective effects than Ac-hE-18A-NH2 or 4F alone, in AD mouse models.

CS-6253, a mimetic peptide derived from the C-terminal lipid-binding domain of APOE, was developed from an earlier version named ATI-5261 [205,206]. CS-6253 has been shown to enhance ABCA1-mediated lipid efflux, promote RCT [206,207], and influence endogenous APO levels in the plasma and to the lesser extend in the brain [208]. It has further been shown that CS-6253 improved APOE lipidation, reduced amyloid and tau pathology, and mitigated APOE4-driven cognitive impairment in mice [163]. In a recent study, Rawat et al. [209] reported that treatment with CS-6253 stabilizes membrane ABCA1 and promotes ABCA1 recycling in primary APOE4 astrocytes. These effects led to increased lipidation and decreased aggregation of APOE4. Furthermore, treatment with CS-6253 enhanced the intracellular Aβ degradation in the presence of recombinant APOE4. These findings corroborate the potential of targeting brain ABCA1 activity using HDL-mimetic peptides to mitigate APOE4-associated neuropathology in AD.

The smallest of the HDL mimetics peptides yet described, CN-105, is five amino acids in length and was derived from the polar face of the receptor-binding region of APOE [210]. CN-105 has higher BBB permeability and potency than longer APOE mimetic peptides, including COG 1410. It has been shown to reduce neuroinflammation and improve survival and functional outcomes in mouse models of ischemic stroke, TBI, and intracranial hemorrhage [210,211,212,213]. A phase 1 randomized, double-blind, placebo-controlled clinical trial of CN-105 showed the peptide was safe and well tolerated in both escalating and multiple dosing regimens, among 48 healthy volunteers [214]. Of note, the plasma half-life of the peptide was determined to be 3.6 h in these patients, significantly longer than that observed for other mimetics. Recently, CN-105 was tested in a mouse model of AD (APP/PS1/APOE4TR mice) and the results showed that amyloid pathology and spatial learning deficits were reduced when the peptide treatment started in younger (14-18 weeks of age) but not older (25-28 weeks of age) mice [215]. This study indicates the importance of initiating treatment early in the course of the disease to achieve functional benefits in AD. Notably, CN-105 has advanced to phase 2 clinical trials for intracerebral hemorrhage (NCT03168581) and postoperative cognitive dysfunction (NCT03802396), raising the possibility of CN-105 as a novel therapeutic agent for AD.

#### 4.2.3. APOJ Mimetic Peptides

Compared to the APOA-I-and APOE-mimetic peptides, APOJ-mimetic peptides are less investigated. The potential of APOJ mimetics to mimic the beneficial aspects of full-length APOJ has been studied. Navab et al. tested a series of small amphipathic peptides derived from APOJ and found that a 10 amino acid mimetic peptide derived from residues (113–122) of the full-length APOJ protein (termed APOJ(113–122)) could inhibit LDL-induced monocyte chemotaxis as effectively as the intact APOJ [216]. Further, it was shown that treatment with APOJ(113–122) in D-amino acids reduced atherosclerosis in APOE-null mice, and improved HDL anti-inflammatory properties in monkeys as well [216]. Importantly, D-APOJ(113–122) had a much longer half-life in the plasma than D-4F, and retained its atheroprotective effects for up to 48 h, after a single dose. APOJ(113–122) has also been shown to reduce inflammation and chemotaxis of primary monocytes derived from patients with systemic lupus erythematosus [217]. Recently, APOJ(113–122) was found to reduce fat accumulation, improve HDL and LDL function, and inhibit atherosclerosis in LDLR-knockout mice [218,219].

The potential of APOJ mimetics in AD has also been explored. In an in vitro study, D-APOJ(113–122) was shown to prevent the formation of Aβ fibrils and promotes the assembly of Aβ into a protective conformation in the presence of an anti-Aβ antibody (scFv-h3D6) without affecting the uptake of Aβ by astrocytes [220]. A follow-up study in a transgenic mouse model of AD (3xTg) showed that treatment with D-APOJ(113–122) by intermittent intraperitoneal (i.p.) injections for 6 weeks reduced Aβ levels in the hippocampus and cortex to a similar extent as treatments with the dual-domain APOE mimetic peptide Ac-hE-18A-NH2 and the anti-Aβ antibody scFv-h3D6 [221]. Interestingly, when the anti-Aβ antibody was combined with either the APOE or APOJ mimetic peptide, the Aβ-reducing effect was interfered rather than enhanced. Further, treatment with each peptide also increased the level of an AD-protective cytokine, interleukin 33 (IL-33), alone or in combination with the anti-Aβ antibody. Interestingly, while the APOE mimetic peptide alone reduced astrocyte and microglial activation, the anti-inflammatory effects of the APOJ mimetic peptide was only observed in combination with the anti-Aβ antibody [221]. In a different mouse model of AD (APP/PS1), work from our own laboratory showed that daily i.p. administration of D-APOJ(113–122) alone for 3 months induced a dramatic decrease in amyloid pathology and rescued memory deficits [222]. In another mouse model of AD (5xFAD), treatment with D-APOJ(113–122) by intraventricular infusion for 2 weeks improved memory function and reduced parenchymal Aβ deposition and CAA [223]. Immunoblot analysis showed an increase in LRP-2 protein in APOJ-peptide-treated brains, suggesting the potential role of LRP-2 in mediating cerebral Aβ reduction. Taken together, the small APOJ mimetic peptide D-APOJ(113–122) presents a promising therapeutic agent for AD.

### 4.3. Other HDL–Enhancing Pharmacotherapies and Strategies

In addition to HDL mimetics, a wide array of HDL-enhancing pharmacotherapies have been developed and are under investigation. These include niacin and niacin receptor agonists, PPARα agonists, CETP inhibitors, liver X receptor agonists and retinoid X receptor agonists, and APOA-I upregulators. These agents are either in clinical use or in clinical trials for cardiovascular disease, while their therapeutic potential for AD has not been thoroughly explored. Interested readers are referred to recent reviews for more discussion on these agents [32,33,224]. Other approaches include immunotherapies targeting non-lipidated, aggregated APOE [225] and gene therapies targeting APOE4 with APOE2 (NCT03634007) [226].

Importantly, non-drug interventions including exercise and lifestyle changes remain the most favorable method of achieving improvements in HDL function to manage cardiovascular and neurological risk. Recent longitudinal studies have shown that adhering to healthy lifestyle behaviors can lower the risk of AD by up to 60% [227]. Although it is well known that exercise and certain diets elevate HDL levels and improve HDL functionality [228,229], the contribution of HDL to the reduced risk of AD in those studies is not clear. Notably, among many lifestyle intervention projects, the National Institutes of Health recently funded the largest exercise research program of its kind, Molecular Transducers of Physical Activity Consortium (MoTrPAC), to study the impact of physical exercise at the molecular level [230]. The results from MoTrPAC are expected to unravel the molecular basis of the health benefits of exercise throughout the body, including metabolism, immune responses, and cardiovascular and brain function, which may provide novel insights into the role of HDL in health and diseases.

Lastly, based on the properties of HDL associated apolipoproteins and HDL mimetic peptides to form lipoprotein complexes and interact with cellular receptors, technologies are emerging to use lipoprotein-based nanoparticles to deliver therapeutic agents across the BBB. The potential of these nanoparticles for the treatment of AD has been explored in several recent studies [157,231,232,233,234]. Interested readers are referred to recent reviews for more discussion on these promising therapeutic approaches for AD [235,236,237,238]. Clearly, HDL-like nanoparticles can serve as carriers for other therapeutics as well as exerting beneficial effects on their own. The specific impact of the HDL-like nanoparticles themselves, versus the drugs they are used to deliver, on AD pathology has yet to be fully elucidated.

## 5. Conclusions

The diverse composition of proteins and lipids that make up HDL particles leads to multifunctionality that exerts benefits beyond the cardiovascular system. Mounting epidemiological, clinical, genetic, and biochemical evidence indicates that peripheral and central HDL metabolism and function influence the risk of AD and its pathogenic course. Recent studies suggest a strong potential for HDL-mimetic peptides to modify the pathogenesis of AD; however, much more research is needed. Clinical studies demonstrating bioavailability and target engagement will be highly important, particularly in the era of antibody-based therapies, which have significant adverse event profiles and well-known limitations in both route of administration and BBB permeability [239]. Also critical is an improved understanding of the safety profile for these HDL mimetics in humans, particularly among elderly people with AD. As APOE4 is the strongest genetic risk factor of AD, reversing APOE4-associated deficits constitutes an attractive approach to defeat this devastating disease. HDL mimetic peptides present a promising tool to achieve this goal.

## Figures and Tables

**Table 1:** Epidemiological and Clinical Evidence for Protective Effects of HDL in Alzheimer’s Disease.

Source	Study Design	Population/Ethnic Group	Study Size	HDL Component	Major Findings
Merched et al., 2000 [35]	Cross-sectional	French	157	APOA-I, HDL-C	Association of low serum APOA-I and HDL-C with AD and poor cognition
Bonarek et al., 2000 [37]	Cross-sectional	French	334	HDL-C	Association of high HDL-C with low risk of AD
Saczynski et al., 2007 [38]	Cohort	Japanese-American	929	APOA-I, HDL-C	Association of high APOA-I with low risk of dementia
Zuliani et al., 2010 [39]	Cross-sectional	Italian	1051	HDL-C	Association of low HDL-C with dementia
Reitz et al., 2010 [40]	Cohort	American	1130	HDL-C	Association of high HDL-C with low risk of probable/possible AD
Song et al., 2012 [41]	Cohort	Australian	664	APOs	Association of low APOA-I, ApoA-II, APOH with high risk of future cognitive decline
Rasmussen et al., 2015 [42]	Cohort	Danish	75,708	APOE	Association of low plasma APOE with increased risk of future AD and all dementia, independent of APOE genotype
Ihle et al., 2017 [43]	Cross-sectional	Brazilian	701	HDL-C	Association of low HDL-C with poor working memory performance
Slot et al., 2017 [44]	Cohort	Dutch	429	APOA-I	Association of low plasma APOA-I with increased risk of clinical progression to AD in APOE4 carriers
Armstrong et al., 2019 [34]	Cohort	American	688	HDL-C	Association of high HDL-C with less steep volumetric decline in entorhinal cortex and parahippocampus
An et al., 2019 [45]	Cohort	Chinese	2514	HDL-C	Association of HDL-C with processing speed and executive function in an inverted U-shaped manner
Svensson et al., 2019 [46]	Cohort	Japanese	1167	HDL-C	Association of high midlife HDL-C with lower risk of late-life MCI and dementia
Chen et al., 2019 [47]	Case-control	Chinese	234	HDL-C	Association of low HDL-C and high TC and LDL-C with AD
Li et al., 2020 [48]	Case-control	Chinese	380	HDL-C	Association of low HDL-C and high LDL-C with AD
Koch et al., 2020 [49]	Cohort	American	1351	HDL/APOE	Association of high APOE level in HDL without APOC3 with better cognitive function and lower dementia risk
Tang et al., 2020 [50]	Case-control (meta-analysis of 27 studies)	Multi-nationality/ethnicity	5286	HDL-C	Association of low HDL-C and high LDL-C with AD in subjects aged 60–70
